# Changes in monoclonal HLA-DR antigen expression in acute organophosphorus pesticide-poisoned patients

**DOI:** 10.3892/etm.2013.1356

**Published:** 2013-10-22

**Authors:** CHENYUN XIA, MI WANG, QI LIANG, LING’AN YUN, HOUSHENG KANG, LEI FAN, DONGSHENG WANG, GUOYUAN ZHANG

**Affiliations:** 1 Affiliated Hospital of North Sichuan Medical College, Nanchong, Sichuan 637000, P.R. China; 2 Department of Nephropathy, People’s Hospital of Peking University, Beijing 100044, P.R. China

**Keywords:** acute organophosphate pesticide poisoning, multiple organ dysfunction syndrome, human leukocyte antigen-DR

## Abstract

The aim of this study was to investigate changes in human leukocyte antigen (HLA)-DR expression of peripheral blood mononuclear cells (MNCs) in patients with acute organophosphorus pesticide poisoning (AOPP). HLA-DR antigen expression of peripheral blood MNCs was examined in 75 patients with AOPP, including 36 patients without multiple organ dysfunction syndrome (non-MODS) and 39 patients with multiple organ dysfunction syndrome (MODS), as well as in 30 healthy individuals using flow cytometry assay. The associations between HLA-DR antigen expression and certain parameters were analyzed, including acute physiology and chronic health evaluation II (APACHE II) score, serum cholinesterase (ChE) activity, cardiac troponin I (cTnI), cardiac enzymes, and liver and kidney function. The mean fluorescence intensity (MCF) of HLA-DR expression in the AOPP group (21.59±5.36) was significantly lower than that in the control group (27.85±4.86) (P<0.001). The MCF in the MODS group (18.17±4.23) was lower than that in the non-MODS group (25.15±6.15). In addition, the MCF of the deceased patients (15.29±3.97) was lower than that of the surviving patients (22.34±2.76) (P<0.001). The MCF of patients with AOPP and MODS was positively correlated with serum ChE (P<0.01) and negatively correlated with the APACHE II score, creatine kinase isoenzyme, cTnI, lactate dehydrogenase, alanine aminotransferase, aspartate aminotransferase, blood urea nitrogen and serum creatinine (P<0.05). In conclusion, HLA-DR expression in patients with AOPP was significantly decreased compared with that in healthy individuals; HLA-DR expression may therefore be a good indicator for evaluating AOPP, MODS disease severity, immune function, efficacy of prognosis and prognosis. Examination of HLA-DR antigen expression may be of crucial clinical value.

## Introduction

Acute organophosphorus pesticide poisoning (AOPP) is a global threat to human health. According to a report by the World Health Organization (WHO) (1), ~3,000,000 people worldwide are affected by pesticide poisoning each year, with AOPP being the most common type. Although an effective antidote for AOPP may be administered, severe cases are likely to lead to the development of multiple organ dysfunction syndromes (MODS). It has been found that the inhibition of cholinesterase (ChE) activity by organic phosphorus in patients with AOPP induces the accumulation of large amounts of acetylcholine within the body, causing cholinergic system dysfunction, hypoxia, inadequate tissue perfusion, microcirculation dysfunction, disseminated intravascular coagulation (DIC) and, ultimately, MODS (2). Human leukocyte antigen (HLA)-DR, the most important effector molecule in antigen presentation in the monocyte-macrophage system, is crucial during specific T lymphocyte immune responses. CD4^+^ T lymphocytes are capable of combining with peptide-loaded HLA-DR molecules on the surface of monocytes or macrophages, initiating T-cell activation and proliferation. HLA-DR expression on the surface of mononuclear cells (MNCs) is closely associated with immune function (3). In addition, previous studies have indicated that HLA-DR expression is associated with the immune state (4,5). However, the role of HLA-DR antigen expression in patients with AOPP and MODS has yet to be elucidated. To date, there have not been any studies indicating that HLA-DR is involved in the occurrence and development of AOPP and MODS. The aim of this study was to explore the correlations between HLA-DR expression and AOPP-associated parameters in order to evaluate their roles in clinical applications.

## Materials and methods

### Patient data

From January 2003 to August 2009, 75 patients were admitted to the emergency room, nephropathy department and intensive care unit of Affiliated Hospital of North Sichuan Medical College (Nanchong, China), having ingested 10–380 ml organophosphorus pesticides. These 75 patients met the diagnosis of AOPP. There were 28 cases of methamidophos poisoning, 15 cases of dichlorvos poisoning, 13 cases of omethoate poisoning, 11 cases of phorate poisoning, five cases of parathion poisoning and three cases of rogor poisoning. There was a time-period of <6 h from poisoning to treatment in all cases. None of the patients had complications from diseases of the heart, brain, liver, lungs or kidneys, or from diabetes, hypertension, malignant tumors or connective tissue diseases. In accordance with the diagnostic criteria for MODS (6) proposed by the American College of Chest Physicians and Critical Care Medicine (ACCP/SCCM) in 1991, the patients were divided into a MODS group (39 cases) and a non-MODS group (36 cases). Patients were scored according to their disease severity by means of the acute physiology and chronic health evaluation II (APACHE II) (7), with the lowest score being 13 points, the highest score being 38 points and the average score being 23.5 points. The study was approved by the hospital Medical Ethics Committee of Affiliated Hospital of North Sichuan Medical College and informed consent was signed by all patients. There were 30 healthy individuals in the control group, which included nine males and 21 females with an age range of 22–65 years and a mean age of 33.5 years.

### Flow cytometry

Peripheral blood samples were collected on admission to the hospital using ethylenediamine tetraacetic acid (EDTA) anticoagulant tubes. Fluorescein isothiocyanate (FITC)-conjugated anti-human HLA-DR monoclonal antibodies (Becton-Dickinson Company, Franklin Lakes, NJ, USA) were used to detect HLA-DR expression according to the manufacturer’s instructions. The mean fluorescence channel number (MCF) was used to measure the expression of HLA-DR.

### Biochemical analysis

Liver function, renal function and creatine kinase levels were examined using a Beckman CX 7 Automatic analyzer (Beckman Coulter Inc., Brea, CA, USA). Cardiac troponin I (cTnI) levels were assessed using a chemiluminescent microparticle immunoassay (CMIA) with a Beckman ACCESS autoimmune luminescence analyzer (Beckman Coulter Inc.). Creatine kinase isoenzyme (CK-MB) levels were examined using a Nissan 7170S automatic chemical analyzer (Nissan, Tokyo, Japan). Serum ChE activity was assessed using the dibutyryl thiocholine method, with reagents provided by Biological Engineering Co., Ltd. of Zhejiang Eastern Europe (Zhejiang, China).

### Statistical analysis

SPSS 13.0 statistical software (SPSS, Inc., Chicago, IL, USA) was used for data analysis and processing. Measurement data are expressed as the mean ± standard deviation. Comparisons between the two groups were conducted using a t-test, and correlation tests were performed using a linear correlation analysis. P<0.05 was considered to indicate a statistically significant difference.

## Results

### Clinical data

Of the 75 patients with AOPP, there were eight deaths from complications of MODS. The mortality rate was 10.7%. All 36 patients in the non-MODS group recovered (Table I).

### Comparisons of HLA-DR antigen expression levels among the groups

**Figure 1:**
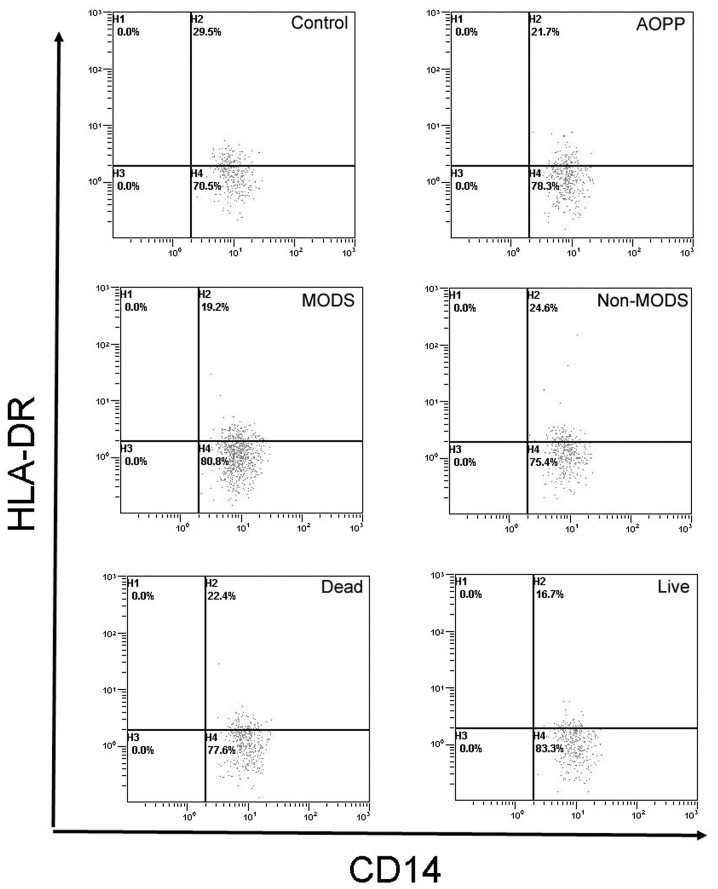
HLA-DR antigen expression levels. The HLA-DR antigen expression level in the AOPP group was 21.7%, which was lower than that in the normal control group (P<0.001). The HLA-DR antigen expression level in the MODS group was lower than that in the non-MODS group (P<0.001). There were no significant differences between the non-MODS group and the normal control group for HLA-DR antigen expression (P>0.05). HLA-DR, human leukocyte antigen-DR; AOPP, acute organophosphorus pesticide poisoning; MODS, multiple organ dysfunction syndrome; non-MODS, non-multiple organ dysfunction syndrome.

HLA-DR antigen expression levels in the AOPP group were lower than those in the normal control group (P<0.001). The levels in the MODS group were lower than those in the non-MODS group (P<0.001). There were no significant differences between the non-MODS and the normal control groups (P>0.05) (Fig. 1).

### Correlation of HLA-DR antigen expression levels with APACHE II score

The APACHE II score (data not shown) of the patients who died in the AOPP group (30.2±7.7) was significantly higher than that of those who survived (22.7±9.7) (P<0.05). HLA-DR antigen expression levels in the patients who died in the AOPP group were significantly lower than the levels in those who survived (P<0.001).

### Correlation of HLA-DR antigen expression levels with clinical indicators

The HLA-DR antigen expression level was positively correlated with serum ChE (r=0.52, P<0.01) and negatively correlated with the APACHE II score, CK-MB, cTnI, lactate dehydrogenase (LDH), alanine aminotransferase (ALT), aspartate aminotransferase (AST), blood urea nitrogen (BUN) and serum creatinine (Scr) (r=−0.61~−0.29, P<0.01 or P<0.05) (Table II).

## Discussion

HLA-DR antigen is one of the class II major histocompatibility (MHC) antigens, mainly expressed in B lymphocytes, monocytes, macrophages, dendritic cells, vascular endothelial cells and activated T cells. It is also expressed in gastrointestinal epithelial cells (8). HLA-DR is crucial for immune system function. In normal circumstances, macrophages, monocytes and other antigen-presenting cells (APCs) engulf and process foreign microbial or exogenous protein into peptides presented on MHC-II molecules, which is capable of initiating an immune response through binding with T helper cells. If HLA-DR antigen expression levels decrease, or its antigen-presenting role is hindered, an effective immune response is not able to be produced (9,10). Thus, the body is not able to effectively remove pathogens and inflammatory mediators. Liao *et al* evaluated the HLA-DR antigen expression of peripheral blood monocytes in patients with severe multiple trauma, demonstrating significant correlations between antigen expression and trauma severity and prognosis. Wang *et al* observed the HLA-DR expression of monocytes in 32 patients with trauma, and found that HLA-DR expression decreased after one day, reached its lowest level on day four, and then gradually recovered. HLA-DR expression in patients with trauma was significantly negatively correlated with the APACHE II score. A sustained decrease in HLA-DR expression in monocytes was associated with a poor prognosis in patients with sepsis, which was one crucial reason for post-injury complications following severe trauma. Tschoeke and Ertel (11) and Cheron *et al*(12) reported that HLA-DR antigen expression decreased among patients with severe pancreatitis. HLA-DR expression gradually returned to normal levels in surviving patients, and progressively decreased in patients who ultimately died; expression levels in these patients were closely associated with mortality (13). The present study showed that the HLA-DR antigen expression of peripheral blood MNCs in the AOPP group was lower than that in the normal control group (P<0.001). HLA-DR antigen expression in patients with AOPP and MODS was lower than that in patients in the non-MODS group. HLA-DR antigen expression was positively correlated with serum ChE, reflecting the degree of AOPP, and was negatively correlated with the APACHE II score (P<0.01), indicating that monocyte HLA-DR antigen expression may be involved in the pathogenesis of AOPP, and may be used as a clinical indicator reflecting AOPP severity. HLA-DR antigen expression levels in patients with MODS were lower than those in patients in the non-MODS group (P<0.01), and were negatively correlated with CK-MB, cTnI, LDH, ALT, AST, BUN and Scr (P<0.01 or P<0.05). This demonstrated that HLA-DR antigen expression of monocytes was closely associated with MODS subsequent to AOPP, which may be an important supplementary mechanism of MODS caused by AOPP. It has been shown that organophosphorus pesticides stimulate the release of cytokines, such as interleukin-1, interleukin-6 and tumor necrosis factor (14), which may increase HLA-DR expression. Endotoxin has been shown to inhibit γ-interferon-induced HLA-DR antigen expression (15). Among patients with AOPP, tumor necrosis factor and endorphin levels may be significantly increased (14), both of which are involved in negative regulation of monocyte HLA-DR antigen expression (16). As HLA-DR antigen expression is reduced, the antigen presenting function becomes impaired (17–20). APACHE II score is positively correlated with disease severity, and is extensively used in monitoring disease conditions and estimating prognosis. In a postoperative study of 30 critically ill patients, Lekkou *et al*(20) observed that monocyte HLA-DR expression was lower in these patients than that in the normal control group. The present study showed that the APACHE II score among those who died in the AOPP group was significantly higher than that among those who survived (P<0.05), and that the monocyte HLA-DR antigen expression level among those who died in the AOPP group was significantly lower than that among those who survived (P<0.01). The APACHE II score of patients with AOPP was significantly negatively correlated with the HLA-DR antigen expression level (P<0.01), indicating that immune function inhibition becomes more apparent with increasing severity of the disease (16). The dynamic observation of monocyte HLA-DR antigen expression levels at different stages may be beneficial in predicting AOPP severity and prognosis. However, in a study of patients with multiple trauma and systemic inflammatory response syndrome by Ploder *et al*(21), HLA-DR antigen expression of monocytes was decreased in all patients compared with the expression levels in the healthy controls, and there was no difference between the group of patients that died and the group that survived. The reasons for the difference between the studies may be associated with different underlying diseases, sample size, genetic background and examination methods. Studies of HLA-DR in AOPP are relatively rare, and the pathogenesis of AOPP and MODS requires further discussion.

## Figures and Tables

**Table I:** Clinical data and HLA-DR antigen expression level in different groups.

		Gender (n)				
					
Group	Cases (n)	Male	Female	Age (years)	HLA-DR antigen expression (MFI)	T-value
Healthy	30	9	21	33.5±11.6	27.85±4.86	5.549^a^
AOPP	75	28	47	34.5±11.3	21.59±5.36	
Non-MODS	36	13	23	33.5±10.2	25.15±6.15	5.764^a^
MODS	39	15	24	35.5±13.6	18.17±4.23	
Surviving	67	25	42	34.1±10.5	22.34±2.76	6.510^a^
Deceased	8	3	5	37.5±12.2	15.29±3.97	

a P<0.01. Data for age are presented as the mean ± standard deviation. HLA-DR, human leukocyte antigen-DR; MFI, mean fluorescence intensity; AOPP, acute organophosphorus pesticide poisoning; non-MODS, non-multiple organ dysfunction syndrome; MODS, multiple organ dysfunction syndrome.

**Table II:** Correlations of HLA-DR antigen with other indicators in patients with AOPP.

Indicators	r	Indicators	r	Indicators	r
APACHE II score	−0.61^b^	CK-MB	−0.51^b^	AST	−0.45^b^
ChE	0.52^b^	LDH	−0.46^b^	BUN	−0.29^a^
cTnI	−0.49^b^	ALT	−0.41^b^	Scr	−0.35^b^

a P<0.05,

b P<0.01.

HLA-DR, human leukocyte antigen-DR; AOPP, acute organophosphorus pesticide poisoning; ChE, serum cholinesterase; cTnI, cardiac troponin I; CK-MB, creatine kinase isoenzyme; LDH, lactate dehydrogenase; ALT, alanine aminotransferase; AST, aspartate aminotransferase; BUN, blood urea nitrogen; Scr, serum creatinine; APACHE II, acute physiology and chronic health evaluation II.
